# Simulation insights on the compound action potential in multifascicular nerves

**DOI:** 10.1371/journal.pcbi.1013452

**Published:** 2025-09-12

**Authors:** Joseph James Tharayil, Ciro Zinno, Filippo Agnesi, Bryn Lloyd, Silvia Farcito, Antonino Cassara, Niels Kuster, Michael Reimann, Silvestro Micera, Esra Neufeld

**Affiliations:** 1 Blue Brain Project, École Polytechnique Fédérale de Lausanne (EPFL) Campus Biotech, Geneva, Switzerland; 2 Foundation for Research on Information Technologies in Society (IT’IS), Zurich, Switzerland; 3 BioRobotics Institute, Sant’Anna School of Advanced Study, Pisa, Italy; 4 Federal Institute of Technology (ETH) Zurich, Zurich, Switzerland; 5 Translational Neuroengineering, EPFL Campus Biotech, Geneva, Switzerland; Institute of Computer Science Czech Academy of Sciences: Ustav informatiky Akademie ved Ceske Republiky, CZECHIA

## Abstract

We developed an extended reciprocity theorem approach to model neuron signals which handles heterogeneous dielectric environments and arbitrary electrode shapes, and use it to study analytically the single fiber action potential. We then established a semi-analytic model that also uses hybrid electromagnetic-electrophysiological simulations to model evoked compound action potential (eCAP) signals from multi-fascicular nerves populated with heterogeneous fiber populations. For validation, model predictions were compared with cuff electrode recordings of activity induced by vagus nerve stimulation in *in vivo* porcine experiments. The semi-analytic model produces signals that approximate the shape and amplitude of *in vivo* measurements. It can account for the important variation in the recorded eCAP due to changes in the shape and placement of the stimulus and recording electrodes. We find that partially activated fascicles contribute particularly to the signal, as eCAP contributions from smoothly varying fiber calibers in fully activated ones partially cancel. As a result, eCAP magnitude does not depend monotonically on the stimulation current and recruitment level. Our method can be used to rapidly assess new stimulation and recording setups involving complex nerves and neurovascular bundles, e.g., to maximize signal information content, for closed-loop control in bioelectronic medicine applications, and potentially to non-destructively reconstruct structural and functional nerve topologies through inverse problem solving. In a proof-of-concept study, we demonstrate that parameter optimization can recover the ground-truth distribution of fiber diameters in a simplifed variant of our model.

## Author summary

The evoked compound action potential (eCAP) is the recorded neural signal that is generated by electrical stimulation. In the context of bioelectronic medicine and neuroprosthetics, the eCAP can be used for monitoring, control, and optimization of stimulation parameters. However, interpretation of the eCAP is non-trivial. In this paper, we develop a computational model of the eCAP in a porcine vagus nerve, and show that this model replicates experimental results. We demonstrate that interpretation of the eCAP is complicated by the structure of the vagus nerve; the signal is sensitive to both the degree to which nerve fibers in particular fascicles are activated, as well as the relative location of those fascicles with respect to the recording electrodes. Partially activated fascicles contribute particularly to the eCAP, while the contributions to the eCAP from individual nerve fibers partially cancel out in fully-activated fascicles, resulting in a non-monotonic dependence of the eCAP amplitude on the degree of fiber activation. A proof-of-concept shows that eCAPs can help infer otherwise inaccessible fiber population statistics crucial for targeted stimulation. In the future, our model may be useful in developing new vagus nerve stimulation and sensing devices and for closed-loop control.

## 1. Introduction

The compound action potential (CAP) is the electric signal that can be picked-up near a nerve and results from propagating axonal action potentials (APs) [1]. It can readily be recorded from a cuff electrode, making it valuable for neural code elucidation, neural sensing, and closed-loop control applications in the field of bioelectronic medicine (‘electroceuticals’, i.e., neural interfaces for regulating (organ-)physiological function, and neuroprosthetics). However, frequently only qualitative changes in, e.g., electrically evoked CAP (eCAP) magnitude are interpreted and the detailed eCAP shape and quantitative features are not leveraged. It is, for example, assumed that eCAP magnitude is monotonically related to the level of axonal recruitment [2].

Vagus nerve stimulation (VNS) is a promising bioelectronic medicine technique, which has already been approved for epilepsy and depression, and is under investigation for many other conditions [3]. Of particular interest are potential applications of VNS to cardiac disorders, including cardiac arrest, heart failure, and arrythmias [4].

The HORIZON 2020-funded NeuHeart project was launched with the aim of restoring autonomic control over cardiac function after heart transplantation with the help of a regenerative neural interface on the cardiac branch of the vagus nerve (VN), which both facilitates re-connection of the heart to the autonomic nervous system and permits to stimulate and regulate corresponding neural input [5]. NeuHeart also includes the development of a series of sensors for closed-loop stimulation control [6]. Computational modeling of implantable neural interfaces plays a central role in designing them, optimizing stimulation parameters, establishing model-based control strategies (where it is combined with an advanced cardiovascular regulation model [7–9]), and interpreting neural signals. eCAP signals are of interest to NeuHeart because 1) they can be used to validate model predictions on fiber recruitment by the implanted stimulation electrodes and 2) they could provide valuable additional feedback for closed-loop control.

Several computational models of the compound action potential have previously been developed. Wijesinghe et al. [1] developed an analytical approach for the calculation of the CAP signal, but it assumes that the axon and nerve are cylindrical and infinitely long, and surrounded by an infinite, homogeneous, and isotropic bath. This makes it impossible to study the impact of internal nerve structure (fascicles, perineurium, epineurium), anisotropy (high within fascicles), or electrode shape. Others have used FEM [10,11] or the boundary element method (BEM) [12] to overcome this problem, but these methods are computationally expensive, since the number of required simulations scales with the number of current sources.

Reciprocity-based approaches [13], which calculate the recorded voltage at each time step by taking the inner product of the membrane currents and a *lead field* that is produced by applying a virtual current between two recording electrodes, can be used to more efficiently calculate extracellular signals in realistic tissue models. While applications of the reciprocity theorem have in general focused on infinitesimal dipoles—a restriction which prevents the application to spatially extended sources such as nerve fibers or neurons with complex morphologies—the general reciprocity approach has been used previously to calculate the recorded CAP in the cochlea [14,15]. More recently, Eiber et al. [16] and Couppey et al. [17] developed Matlab- and Python-based pipelines, respectively, to calculate electrical-stimulus-evoked compound action potentials in peripheral nerves, using reciprocity-based approaches to simulate electrical recordings. BlueRecording [18], a set of tools integrated with NEURON, and Sim4Life (Zurich MedTech AG), a commercial FEM software package, also use reciprocity-based methods to simulate electrical recordings.

While these approaches are both efficient and accurate, without access to high-performance computing resources, it remains computationally infeasible to simulate the full number of nerve fibers (over 300,000 in the vagus nerve [19]) and their heterogeneity (myelinated/unmyelinated, different fiber diameter probability distributions across the nerve topology). Goldstein and Kiang [20] developed a simplified model of the CAP, where the CAP is the convolution of a firing rate function with the waveform of the single-fiber action potential (SFAP, i.e., the contribution to a recorded extracellular signal from by an action potential in a single fiber). Variations of this model have been applied, for example, to model the auditory nerve [21–24], but they are limited by the assumption that the SFAP shapes all contribute in a similar manner to the CAP signal—thereby limiting the ability of these models to account for the sensitivity of the eCAP to recording electrode placement relative to the fibers—and by the need to empirically estimate the SFAP shape.

As an alternative to simulating fibers individually, Pe na et al. recently published a semi-analytic model of the eCAP [25], which leverages a similar approach to the one we introduced in [26]. This method involves calculating transmembrane currents during an action potential from a small number of fibers, convolving these currents with an “exposure function” that accounts for the lead field as well as for diameter-dependent SFAP amplitude and conduction velocity, and summing over the distribution of fiber diameters. Pe na et al. validated this approach in a model of the rat cervical vagus nerve. However, the rat vagus nerve, unlike that of the pig and the human, contains only a single fascicle. Moreover, Pe na et al. assume that all fibers in the model are activated by the stimulus. Here, we extended this approach to multi-fascicular nerves with realistic, heterogeneous afferent and efferent, myelinated and unmyelinated fiber populations and spatially varying diameter probability distributions and consider the effects of realistic recruitment curves on the recorded signal. We show that these factors critically influence the eCAP. This refined model is applicable to studying complex, multi-fascicular nerves and neurovascular bundles. The results were experimentally validated.

The goals of this study were to:

- develop the *extended reciprocity theorem* approach as analytical basis for the accurate and efficient modeling of neural signals resulting from spatially extended neural activities in models of complex nerves with realistic neural interface designs;
- build a realistic semi-analytic model of the cardiac VN to support the NeuHeart project;
- compare predictions from that model against data from *in vivo* porcine experiments; and
- leverage the developed methodologies to improve understanding of the eCAP signal.

**Fig 1:**
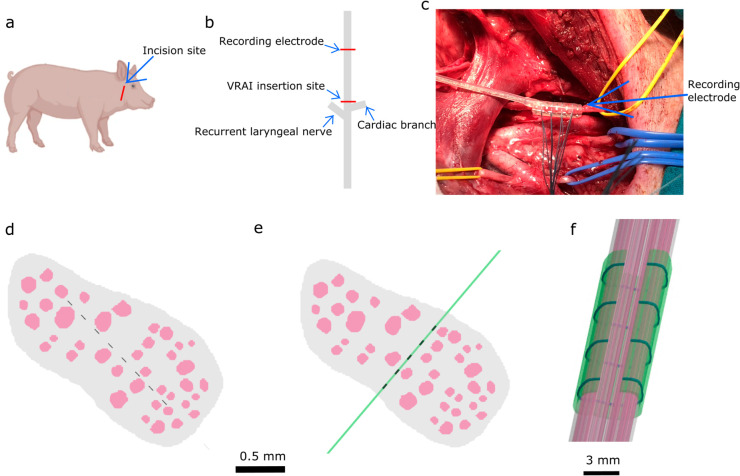
In vivo and in silico experimental setups. a: Incision site on the minipig. (adapted from [28] under a CC-BY 4.0 license). b: Schematic of the VN. c: Photo taken during surgery, showing the recording electrode on the VN. d-e: VN model with stimulation electrode in horizontal (d) and vertical (e) orientations. Note that the histology-based cross section topology likely differs from that of the experimentally stimulated nerve (different minipigs). In the horizontal orientation, the insulating substrate (green element in subpanel e) is not included, to avoid piercing fascicles. As all stimulus contacts (black rectangles) are active, this does not significantly influence the E-field. f: VN model with recording cuff electrode. Recording contacts (blue) are surrounded by insulation (green).

More specifically, we stimulate the vagus nerve with the intrafascicular NeuHeart Vagal Regenerative Autonomic Interface (VRAI) electrode (Fig 1A–1B, 1D–1E, [27]) and record eCAP signals with a cuff electrode (Fig 1A–1C, 1F). We compare eCAPs recorded in *in vivo* experiments in minipigs (Fig 1A–1C) with *in silico* eCAPs calculated using our vagus nerve (Fig 1D–1F).

## 2. Results

The results section of the paper is organized as follows: First, we fit analytical formulae to the relationship between fiber diameter and velocity, and between fiber diameter and stimulus threshold. Next, we present our *analytical model* of the SFAP, described in Sect 5.2.2, which predicts the SFAP as a function of action potential shape and propagation velocity, and of the recording electrode potential, all of which are calculated numerically. We validate the analytic model against other approaches in simplified dielectric environments. We then present a *simplified analytical model*, described in Sect 5.2.4, in which the action potential shape and recording electrode potential are approximated by closed-form functions. We analyze the relationship between the SFAP and the fiber diameter and inter-electrode spacing under these simplifying assumptions. Next, using realistic AP and lead-field shapes, we calculate the stimulus-triggered eCAP with a *semi-analytic model* (described in Sect 5.2.6), which predicts the eCAP based on numerically computed characteristics of fiber models (rather than the closed-form functions used in the analytical model), an electromagnetic (EM) simulation of current applied to the recording electrodes, histology derived distributions of different fiber populations, and fiber recruitment curves obtained through coupled EM-electrophysiologial simulations. We compare the results of our semi-analytic model to *in vivo* experimental results, and assess fascicular contributions to the eCAP as a function of stimulation and recording electrode geometry. Finally, we show that by optimizing the parameters of our semi-analytic model, we can recover the fiber diameter distribution in a nerve based on the recorded eCAP, thus solving the inverse problem.

### 2.1. Fitting of fiber model

**Fig 2:**
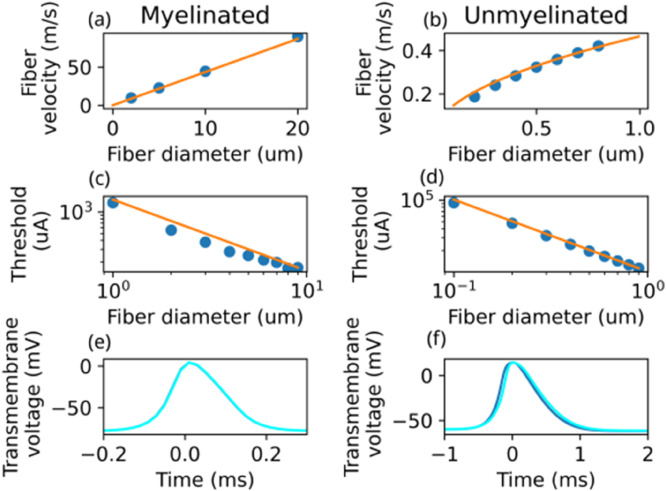
Properties of the fiber models used in this study. a-d: Blue dots indicate data points extracted from electrophysiological simulations; orange lines indicate fits to theoretical relationships. First row: Fiber velocity depends linearly on diameter for myelinated fibers (panel a) and is proportional to the square root of velocity for unmyelinated fibers (panel b). Second row: Stimulus threshold is inversely proportional to fiber diameter for myelinated (panel c) and unmyelinated (panel d) fibers. Third row: The temporal shape of the AP does not vary significantly with diameter for myelinated fibers (panel e; light blue: d=10μm, dark blue: d=20μm) and for unmyelinated fibers (panel f; light blue: d=0.4μm, dark blue: d=0.8μm).

For myelinated fibers, conduction velocity is proportional to diameter [29], while for unmyelinated fibers, conduction velocity is proportional to the square root of diameter [30]. These relationships were reproduced with the electrophysiological models used in this study (see Fig 2). For the myelinated fiber model, the proportionality constant a=d/v was determined to be $\sim 4.3\;\cdot \;10^{6}s^{-1}$ (determined for a 20 *μ*m fiber). For the unmyelinated fiber model, the proportionality constant $b=\sqrt{d}/v\approx 470m^{0.5}s^{-1}$ (based on a 0.8*μ*m fiber). The width of the action potential (or more precisely, the width of a Gaussian fit to the action potential shape) is $5\;\cdot \;10^{-5}$s for a myelinated fiber (Fig 2C) and $2\;\cdot \;10^{-4}$s for an unmyelinated fiber (Fig 2D). For both myelinated and unmyelinated fibers, we confirmed that the stimulus threshold is proportional to $\frac{1}{d}$ (Fig 2C–2D).

### 2.2. Validation of the analytical model of the SFAP

According to the reciprocity theorem [13], the voltage signal (*S*) recorded between two electrodes resulting from a dipolar current source $\vec{d}$ is related to the electric (E-)field $\vec{E}$ that is generated at the dipole location by (virtually) applying a current *J* to the same electrodes:

$$J\cdot S=-\vec{E}\cdot \vec{d}.$$ (1)

For a neural fiber with a transmembrane current density *i*(*l*) along its length, and given that $\vec{E}=-J\nabla \Phi$ Eq 1 can be expressed (c.f. Sect 2.2) as the *extended reciprocity theorem*:

$$S=\int_{0}^{l}\Phi (l^{\prime })i(l^{\prime })dx^{\prime }.$$ (2)

We call Φ(l) the “sensitivity function”; it represents the potential field that is generated by a virtual unit current applied to the recording electrodes. For an action potential that propagates with velocity *v*, the signal is given by:

S(t)=∫i(l−tv)Φ(l)dl. (3)

The spatial profile of the transmembrane current, *i*(*l*), is proportional to the second derivative of the temporal profile of the transmembrane current. During an action potential, Eq 3 can be expressed (c.f. Sects 5.2.1–5.2.2) as:

$$S(t)=\frac{\eta_{\tau }}{v}(\partial_{t}^{2}V_{t}(t)*\Phi (v\cdot t)),$$ (4)

where $V_{t}$ is the waveform (at a given location, as a function of time) of the transmembrane potential during an AP (Fig 2C–2D), $\eta_{\tau }$ is a proportionality constant that depends on the fiber diameter and the electrical properties of the fiber, and ‘*’ signifies convolution in time.

**Fig 3:**
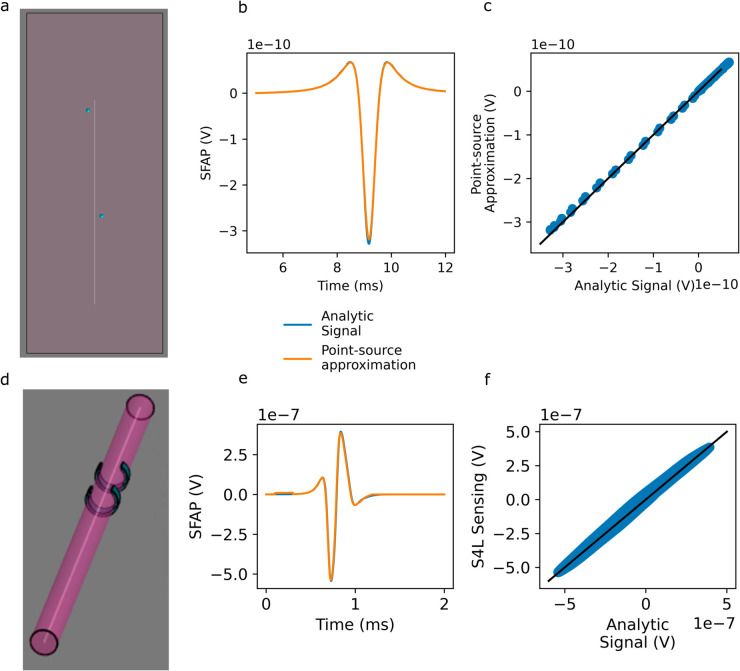
Verification of the analytical SFAP calculation. a: Large homogeneous environment used for calculations in panel b. Locations of recording electrodes are marked by blue spheres (enlarged for visualization). b: SFAPs calculated in a large homogeneous volume using analytic method compared to reference result obtained using point-source approximation. c: Correlations between predicted and reference voltages in (b). The signals are in good agreement (*R*^2^ = 0.99, slope=0.97, intercept=10^−13^). The black line represents perfect agreement. d: Simplified nerve geometry used for the calculations in panel (e). e: SFAP calculated using the analytical method compared to the reference result obtained using the Sim4Life Neural Sensing tool. f: Correlations between predicted and reference voltages in (e). The signals agree well (*R*^2^ = 0.99, slope=0.98, intercept=2 10^−10^). The black line represents perfect agreement.

To validate our approach, we calculate the SFAP of a myelinated fiber using the point-source approximation in a large homogeneous environment (Fig 3A) and using the Sim4Life Neural Sensing Tool in a simplified nerve geometry (Fig 3D), and compare the results to the SFAP calculated using Eq 4, for which we calculate Φ numerically and obtain $V_{t}$ and *v* numerically from a Rat [31] fiber model. The SFAP calculated using our method agrees well with that calculated using the point-source method (Fig 3B–3C). In a simplified nerve model (Fig 3D), the SFAP calculated using our analytic method aligns well with that calculated using the Sim4Life Neural Sensing tool (Fig 3E–3F; note that between 0.1 and 0.3 ms, Fig 3E shows a stimulus artifact which appears in the SFAP from the Neural Sensing Tool but not in the analytically-calculated SFAP). These verification benchmarks confirm the suitability and implementation of our analytic approach.

The neural simulation requires ∼40 seconds to run in Sim4Life, for a single neuron. Simulating the entire vagus nerve, with over 300000 neurons, would therefore require 3000 core-hours. In comparison, simulating the nerve using the semi-analytic method requires 0.66 core-hours, thus providing a factor ×5000 speedup. Moreover, the point-source approximation cannot handle inhomogeneous dielectric environments, while our analytic approach readily does.

### 2.3. Analysis of SFAP shape using a simplified analytical model

To qualitatively analyze the analytical model in Sect 2.2, we approximate the shape of the action potential and of the sensitivity function from a monopolar electrode at position *x*_*c*_ as Gaussians. The constants $V_{p}$ and $w_{V}$, denote the amplitude and width, respectively, of the Gaussian approximation of the action potential, and $\Phi_{p}$, $w_{\phi }$ and *x*_*c*_ the amplitude, width, and center of the Gaussian approximation of the sensitivity function. These approximations are only used for the analytical investigation reported in this section, in order to allow for a closed-form solution to the SFAP, while realistic shapes for the AP and sensitivity function are used elsewhere. We obtain

$$S(t)=\frac{V_{p}\Phi_{p}\eta_{\tau }w_{\phi }w_{V}}{w_{C}^{5}v^{2}}e^{-\frac{{\left(\frac{x_{c}}{v}-t\right)}^{2}}{2w_{C}^{2}}}\left(t-\left(\frac{x_{c}}{v}+w_{C}\right)\right)\left(t-\left(\frac{x_{c}}{v}-w_{C}\right)\right),$$ (5)

where $w_{C}=\sqrt{(\frac{w_{\phi }}{v}{)}^{2}+w_{V}^{2}}$ (see Sect 5.2.4 for a full derivation). *w*_*c*_ corresponds to the duration, in time, of the SFAP waveform recorded by a monopolar electrode at location *x*_*c*_. The spatial extent of the SFAP—that is, the maximal distance from *x*_*c*_ where the SFAP can be recorded at the time when the SFAP at *x*_*c*_ is at its peak—is given by $w_{c}v$, where *v* is the propagation velocity of the fiber.

**Fig 4:**
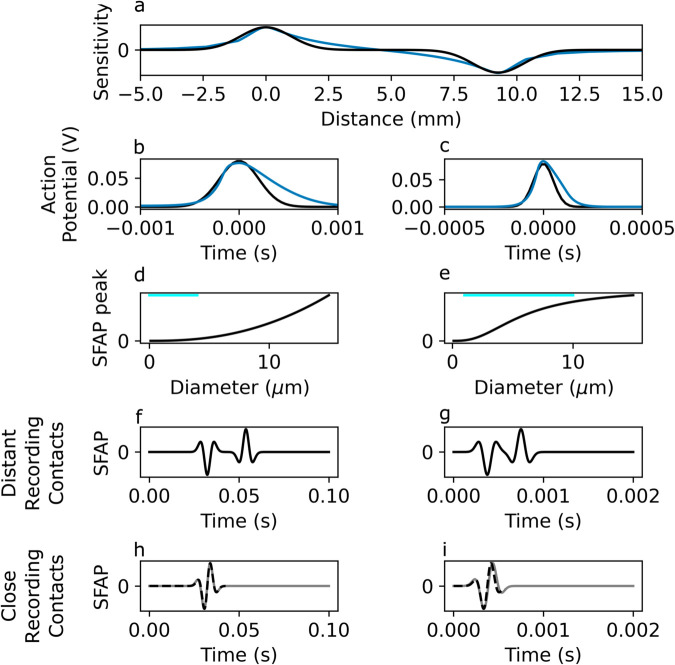
Analytical investigation of the properties and behavior of the SFAP in the simplified model. a: Normalized sensitivity function from a bipolar electrode in a realistic nerve geometry (blue trace) and a Gaussian approximation of a bipolar sensitivity function at the same location (black trace). b, c: Action potential shapes from an unmyelinated (left) and myelinated (right) fiber model (blue traces) and Gaussian approximations of the action potential shapes (black traces). d-i: Results of the analytical investigation. d, e: The SFAP amplitude increases with diameter for unmyelinated fibers (left) but plateaus for the myelinated fibers (right). Light blue bars indicate realistic fiber diameter range. f, g: Distinct trimodal waveforms are apparent for sufficiently separated contacts in the bipolar recordings of an unmyelinated fiber (f; diameter: 1 *μ*m, spacing: 1 cm) and a myelinated fiber (g; diameter: 10 *μ*m, spacing: 1.5 cm). h,i: With decreasing contact separation, the two waveforms merge and a single tetramodal one results (h: unmyelinated fiber, 0.1 cm spacing; i: myelinated fiber, 0.5 cm spacing). As expected, it resembles the derivative of the original waveform (dashed).

Using Eq 5, we calculate the peak amplitude of the SFAP as a function of diameter. For the recording electrodes used in this study, $w_{\phi }\sim 1$ mm (Fig 4A). Propagation velocities *v* and AP width $w_{V}$ are calculated in Sect 2.1.

Because, for realistic unmyelinated fiber diameters, $v<<w_{\phi }/w_{V}$ (c.f. Sect 5.2.4, Table 1), their SFAP contribution magnitude monotonously increases with diameter (Fig 4D). For myelinated fibers, whose velocity is linearly dependent on diameter, however, the contribution weight saturates for large fibers (Fig 4E).

**Table 1:** Velocity as a function of diameter (first column) and diameter *d*  at which $v=\frac{w_{\phi }}{w_{V}}$, for $w_{\phi }=1\;$mm, (second column) for myelinated and unmyelinated fibers. Maximum amplitude of SFAP for myelinated and unmyelinated fibers, in general (third column) and in the two limit cases (AP extent much shorter or longer than electrode potential; fourth and fifth columns). $\Psi =\frac{V_{p}\Phi_{p}\pi \sigma }{4}$.

	*v*	*d*		general	$d\ll d^{*}$	$d\gg d^{*}$
			*w*_*C*_:	$\sqrt{(\frac{w_{\phi }}{v}{)}^{2}+w_{V}^{2}}$	$w_{\phi }/v$	$w_{V}$
myelin.:	$4.3\;\cdot \;10^{6}d$	4.7 *μ*m	amplitude:	$\frac{\Psi w_{\phi }w_{V}}{a^{2}w_{C}^{3}}$	$\frac{\Psi aw_{V}}{w_{\phi }^{2}}d^{3}$	$\frac{\Psi w_{\phi }}{a^{2}w_{V}^{2}}$
unmyel.:	$470\sqrt{d}$	110 *μ*m	amplitude:	$\frac{\Psi dw_{\phi }w_{V}}{b^{2}w_{C}^{3}}$	$\frac{\Psi bw_{V}}{w_{\phi }^{2}}d^{5/2}$	$\frac{\Psi w_{\phi }}{b^{2}w_{V}^{2}}d$

A bipolar electrode can be approximated as the combination of two monopolar contacts at positions *x*_*c*,1_ and *x*_*c*,2_. When the two electrodes are sufficiently separated they produce an SFAP that simply reflects the distinct arrival of the AP at the two electrodes and their opposite polarities (Fig 4F–4G). However, once $\frac{x_{c,2}-x_{c,1}}{v}$ is in the same order of magnitude as *w*_*C*_, the two shapes start to overlap, which can result in fewer lobes. For unmyelinated fibers in the range of 0.1 to 4 *μ*m, the velocity $b\;\cdot \;\sqrt{d}$ ranges from 0.15 to 1 m/s, which in combination with $w_{V}$ results in a spatial AP extent in the order of 1 mm (Fig 4H). For myelinated fibers in the range of 1 to 10 *μ*m, the velocity a·d ranges from 4.4 to 44 m/s, which in combination with $w_{V}$ results in the same spatial AP extent in the order of 1 mm (Fig 4I). This implies that an electrode separation of less than 1 mm will result in the merging of the SFAP waveforms. Because of the bimodal shape of *ϕ* in bipolar recordings, convolution with *ϕ* is akin to a finite difference derivative and produces a signal that resembles the first derivative of a monopolar recording (see Fig 4 and Sect 2.6).

### 2.4. Semi-analytic model replicates shape of *in vivo* eCAP

The NeuHeart VRAI stimulation electrode and a recording cuff electrode were implanted in the vagus nerve of minipigs (Fig 1A–1C), and eCAPs evoked by various stimulus current levels were recorded. Details of the experimental procedure are provided in Sect 5.3.

**Fig 5:**
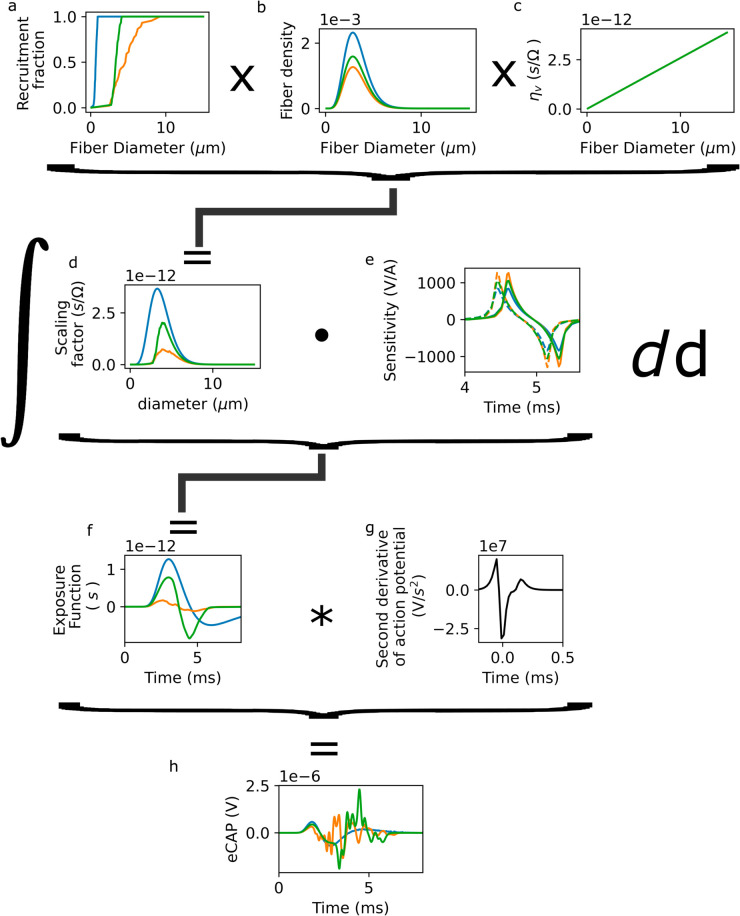
Calculation of the contribution to the eCAP for myelinated afferent fibers from three representative fascicles. a: Fraction of myelinated afferent fibers *R*_*i*_(*d*) recruited as a function of diameter. b: Myelinated afferent fiber diameter distribution $N_{i}\;\cdot \;p(d)$. c: Scaling $\frac{\eta_{\tau }(d)}{v(d)}=\frac{\pi d^{2}\sigma }{4v(d)}$ of transmembrane currents as a function of fiber diameter. d: Scaling factor $R_{i}(d)\;\cdot \;N_{i}p(d)\;\cdot \;\frac{\eta_{\tau }(d)}{v(d)}$. e: Sensitivity function $\Phi_{i}(d,t)$, plotted for diameters 3 *μ*m (solid lines) and 3.1*μ*m (dashed lines). f: Exposure function *X*_*i*_(*t*) for each fascicle. Sum of Φ(d,t) scaled by the diameter-dependent factor $R_{i}(d)\;\cdot \;N_{i}p(d)\;\cdot \;\frac{\eta_{\tau }(d)}{v(d)}$ over all diameters. g: Second derivative of the AP; identical to the transmembrane current up to a scaling factor $\frac{\eta_{\tau }}{v}$ (panel c). h: eCAP contribution; convolution of f and g.

To model the eCAP from the complete vagus nerve, we develop a semi-analytic model based on the SFAP from Eq 4. The model is described in detail in Sect 5.2.6. For each fascicle, we instantiate a population of fibers with a uniform diameter distribution. We define a “scaling function” $R(d)\;\cdot \;N\;\cdot \;p(d)\;\cdot \;\frac{\eta (d)}{v(d)}$ (Fig 5D), where *R* is the fraction of fibers of diameter *d* that are activated at a given current level (Fig 5A), *N* is the number of fibers in the fascicle, and *p* is the density of fibers in the fascicle for diameter *d* (Fig 5B), *v* is the fiber velocity and *η* the prefactor from Eq 4 (Fig 5C).

To calculate the recruitment we create a 2.5D finite element model of the vagus nerve by extruding a 2D segmented image created from a histological slice and inserting models of the stimulating and recording electrodes (Fig 1D). A population of fibers of uniform diameter *d*_0_ is instantiated in each fascicle, and activation thresholds are calculated using Sim4Life, for a biphasic stimulus pulse with all of the stimulation electrodes active. From these activation thresholds, we determine the recruitment curve *R*(*d*/*d*_0_,*I*), where *I* is the stimulus current amplitude (c.f. Sect 5.4.3).

To calculate the eCAP, we multiply the scaling function by the sensitivity function Φ from our finite element model and reparameterized in terms of v·t (Fig 5E) to produce an “exposure function” (Fig 5F), and convolve the exposure function with the second derivative of the transmembrane potential (Fig 5G), obtained from a NEURON simulation of a Rat [31] or Sundt [32] fiber, for myelinated and unmyelinated fibers, respectively. This procedure is equivalent to scaling the SFAP by recruitment and fiber density, but is more computationally efficient.

**Fig 6:**
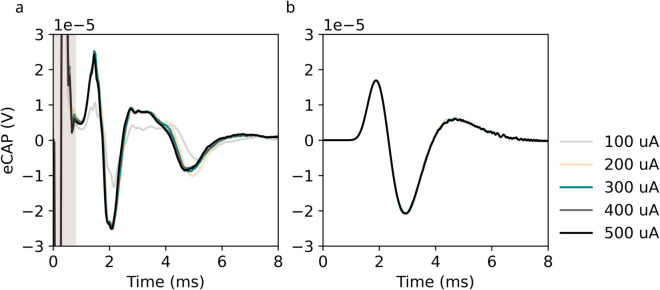
The semi-analytic model replicates the in vivo eCAP. a: *In vivo* eCAP recorded 6 cm from the stimulus electrode. A large stimulus artifact is visible from t=0 to t=0.6 ms (shaded area). b: Semi-analytic model eCAP recorded 6 cm from stimulus electrode.

The semi-analytic model is able to replicate key features of the *in vivo* eCAP, as well as its magnitude. The overall shape of the *in vivo* eCAP (Fig 6A)—with an initial positive deflection, a negative deflection of similar width and magnitude, and a longer, lower-amplitude positive deflection—is reproduced by the model (Fig 6B). However, the second negative deflection at 5 ms is not reproduced. While the model replicates the saturation of the eCAP signal with increasing current, it does not replicate the difference in amplitude between the eCAP evoked at *I* = 100 *μ*A and eCAPs evoked at greater amplitudes. The magnitude of the model eCAP is comparable to that of the *in vivo* signal.

### 2.5. Fascicular contributions to eCAP cluster with degree of activation

To explore the influence of fiber activation on the eCAP, we reduce the stimulus amplitude to 31.25 *μ*A, (i.e., 500 *μ*A total current divided equally over the 16 electrodes), as larger amplitudes result in complete activation of the myelinated fibers in the model. The shape and amplitude of the fascicular contribution to the eCAP depend non-trivially on the degree of activation. For strongly activated fascicles (Fig 5A, blue curve), the exposure function *X*_*i*_(*t*) (Fig 5E), and correspondingly, the eCAP (Fig 5H) produced by convolution with the transmembrane current (Fig 5G) are smooth. For fascicles with an intermediate degree of activation (Fig 5A, orange curve), the exposure function (Fig 5F) is non-smooth, resulting in a large-amplitude deflection in the eCAP at 3.5 ms (Fig 5H). The reason is that the absence of activated fibers below 3 *μ*m prevents cancellation of the contributions of fibers above this threshold are not canceled out (see Sect 3.1).

**Fig 7:**
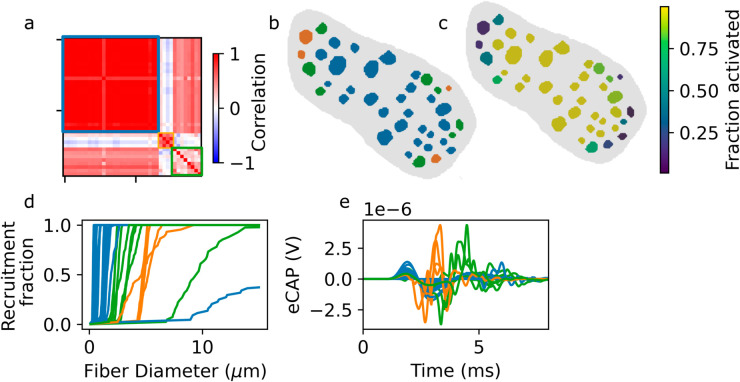
Fascicular recruitment dominates the eCAP contribution shape. a: Correlations between eCAP contributions of the different fascicles (stimulus amplitude: 31.25 *μ*A). Three clusters, identified using k-means, are outlined (cluster color coding consistent throughout Figs 7 and 8). b: Cross section of the nerve, with fascicles colored by cluster. c: Fraction of myelinated fibers activated in each fascicle d: Cluster-colored recruitment curves for individual fascicles. e: Cluster-colored eCAP contributions for individual fascicles.

As a result, the eCAP contributions from the individual fascicles are strongly correlated for fascicles with similar degrees of activation (Fig 7, Pearson product-moment correlation). The fascicles were clustered into three groups based on the correlations between their eCAP contributions, using k-means clustering. Fascicles in each cluster have similar spatial locations (Fig 7B), fractions of fibers activated (Fig 7C), recruitment curves (Fig 7D), and eCAP contributions (Fig 7E). Fascicles in the center of the nerve, close to the stimulus electrode, belong to one cluster with high activation, steep recruitment curves, and smooth eCAP contributions. In contrast, fascicles at the edges of the nerve, farther form the stimulus electrodes, have a lower activation fraction, and consequently, less smooth eCAP contributions.

**Fig 8:**
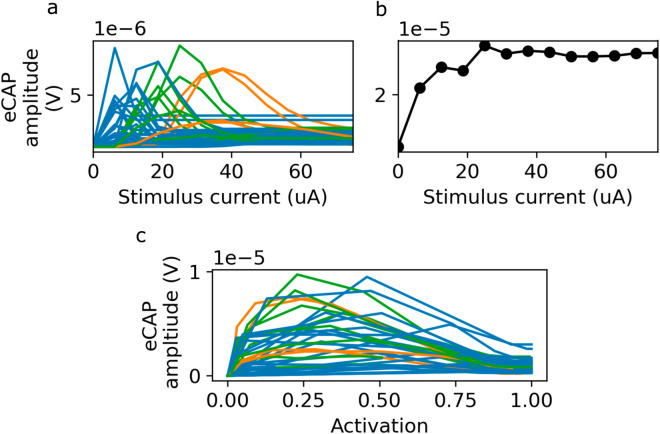
eCAP magnitudes are non-monotonic with respect to recruitment. a: Peak amplitude of the fascicular eCAP contributions, as a function of current. Curves for each fascicle are colored according to the clustering from Fig 7B: Peak amplitude of the total eCAP, as a function of stimulus current. c: As in a, but as a function of their degree of activation.

The stimulus amplitude (*I*) dependent peak magnitude of the fascicular contributions to the eCAP follow a stereotyped pattern, rising to a maximum before decaying again and plateauing once recruitment reaches 100% (Fig 8A). This can be explained by the non-monotonic relationship between fiber activation and eCAP amplitude demonstrated in Fig 5. Consequently, the overall eCAP also fails to monotonically increase with fiber activation (Fig 8B). As expected, fascicles in the same cluster, (see above), have similar stimulus-eCAP amplitude relationships (Fig 8A).

### 2.6. Interactions between fascicular structure and stimulation and recording electrode geometry influence eCAP

**Fig 9:**
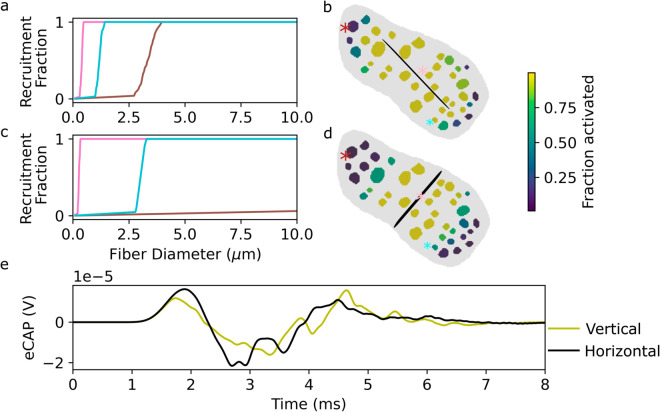
Stimulus electrode orientation influences eCAP due to differential activation of fascicles. a: Recruitment curves for selected fascicles, electrode inserted along the major axis of the nerve (stimulus amplitude: 31.25*μ*A) b: Fraction of myelinated fibers activated for each fascicle (electrode trajectory highlighted with black line for visualization purposes only; selected fascicles from panel a are marked with correspondingly colored asterisks). c-d: Same as a-b, for electrode insertion along the minor axis of the nerve. e: Simulated eCAPs.

We next generate a new finite element model with the stimulus electrode oriented along the minor axis of the nerve (Fig 9D), recompute the recruitment curves *R*_*i*_(*d*), and run the semi-analytical model for this configuration. The insertion angle of the stimulus electrode with respect to the nerve has a substantial impact on the eCAP (Fig 9E), as changes in the distance and orientation of the stimulus contacts relative to the different fascicles substantially change the degree of fiber activation therein (Fig 9B, 9D).

**Fig 10:**
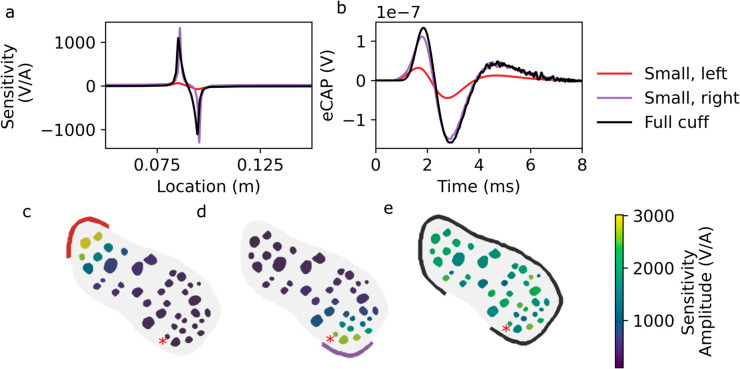
The size and placement of the recording electrode influences the eCAP due to differing fascicular sensitivities $\Phi_{i}$. a: Sensitivity to activity in a selected fascicle (marked with red asterisk in panels c-e) for three different electrodes. b: Corresponding eCAP contributions. c-e: Peak-to-peak magnitude of sensitivity function, for each fascicle and recording electrode configuration.

We also generate new finite element models with the span of the recording electrode reduced, and with varying orientations of this smaller recording electrode (Fig 10C–10E), in order to recompute the sensitivity function Φ(l), and evaluate the semi-analytic model. As was the case for the stimulus electrode, the orientation and extent of the recording electrode with respect to the nerve influences the eCAP (Fig 10B), because the distance from the electrode to each fascicle (Fig 10C–10E) has a significant effect on each fascicle’s sensitivity function $\Phi_{i}(l)$ (Fig 10A).

**Fig 11:**
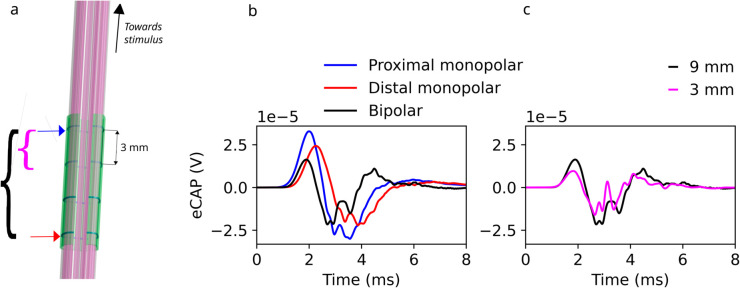
The recording configuration affects eCAPs recorded by a four contact cuff electrode (31.25 *μ*A stimulus current). a: Schematic of recording electrode. Blue and red arrows indicate proximal and distal monopolar recording electrodes. Black and purple brackets indicate pairs of recording electrodes with 9mm and 3mm separation, respectively. b: Monopolar recordings with the most proximal or the most distal contact (distant reference), plotted alongside a bipolar recording using both contacts (9 mm separation). c: Bipolar recordings using 9 vs. 3 mm separation.

We next compare eCAPs recorded using two bipolar cuff electrodes to monopolar recordings using one of the cuff electrodes with a distant reference. Compared to monopolar recordings, bipolar ones with small recording contact separation correspond to a finite difference derivative, such that the number of signal phases with alternating polarity (‘lobes’) is increased by 1—for the simulated eCAP in Fig 11B the monopolar signal is approximately biphasic and the bipolar one triphasic. As the contact separation increases, this corresponds to coarsening and smoothing of the finite difference derivative. It results in reduced fluctuations and noise (Fig 11C), which potentially represents a loss in temporal information content. If the separation is increased further, the signal shape undergoes a complex transformation, until two distinct monopolar recording shapes emerge.

### 2.7. Inference of fiber characteristics

A key advantage of our model-based approach is the ability to draw inferences between the observed features of the eCAP and the underlying biology. A natural extension of our model, therefore, would be to leverage it in order to reconstruct the structural and functional organization of the nerve based on the *in vivo* eCAP signal. This can be accomplished using an optimization approach: by fitting parameters of our model in order to minimize a difference metric between the *in silico* and *in vivo* eCAPs, the biological values of those parameters can be estimated.

**Fig 12:**
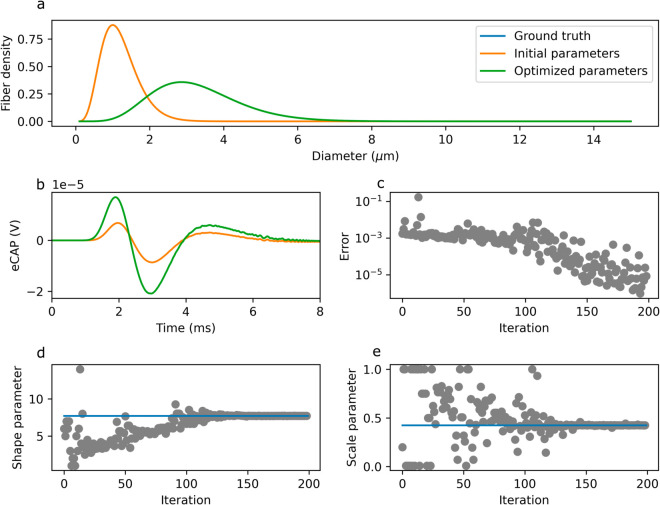
Proof-of-concept of fiber population statistics inference from eCAP data. Optimization is able to recover ground-truth fiber distribution parameters. a: Reference diameter distribution, and diameter distributions with initial and inferred (i.e., optimized) parameters. b: Reference eCAP and eCAPs obtained with initial and inferred (i.e., optimized) parameters. c: Difference metric between reference eCAP and eCAP with inferred parameters decreases sharply after ∼100 optimization iterations. d-e: Shape parameter (d) and scale parameter (e) converge to ground-truth values (indicated by blue line).

As a proof-of-concept, we attempt to recover the shape and scale parameters of the fiber diameter distribution for myelinated afferents, keeping the parameters for the myelinated efferents fixed, and ignoring (for computational efficiency, and without loss of generality) the unmyelinated fibers. Beginning with an arbitrary set of parameters, we optimize them such that the calculated eCAP matches the “ground-truth” eCAP (i.e., that in Fig 6B), for which we know the fiber diameter distribution. Our optimization approach is able to almost perfectly (L1 norm: $9.5*10^{-7}$) reproduce the ground-truth eCAP (Fig 12A) after ∼200 iterations (Fig 12B). The distribution parameters obtained through optimization are nearly identical (Relative error: $4*10^{-4}$ and $1*10^{-4}$ for the shape and scale parameters, respectively) to the ground-truth parameters (Fig 12C–12E).

## 3. Discussions

### 3.1. eCAP cancellation and partial nerve activation

Based on Eq (2), it can readily be shown that eCAP signal contributions subtly annihilate on the single fiber and fiber population level:

#### 3.1.1. Single fiber:

For propagating APs, Eq (2) can be restated as:

$$S(t)=\int i(t-\frac{x}{v})\Phi (x)dx=\frac{\eta_{\tau }}{v^{2}}\int {V_{t}^{\prime }}^{\prime }(t-\frac{x}{v})\Phi (x)dx=\eta_{\tau }\int V_{t}(t-\frac{x}{v})\Phi^{\prime \prime }(x)dx$$ (6)

where *i*(*t*) and *V*(*t*) refer to the time dependent transmembrane current and voltage at reference location *x* = 0.

Different situations can be distinguished: If the AP’s spatial extent is much larger than that of a sensing electrode contact at location *x*_0_ (or, more precisely, that of its *ϕ* along the fiber; see as well Sect 2.3), *S*(*t*) reduces to:

$$S(t)\approx \frac{\eta_{\tau }}{v^{2}}{V_{t}^{\prime }}^{\prime }(t-\frac{x_{0}}{v})\int (\Phi (x)-\Phi_{\infty })dx=\int (\Phi (x)-\Phi_{\infty })dx\;\cdot \;i(t-\frac{x_{0}}{v}),$$ (7)

which is proportional to the time-varying transmembrane current at the electrode location (see Sect 5.2.5 for full derivation). This is a common situation for myelinated fibers.

Similarly (see Sect 5.2.5), when the AP’s spatial extent is much shorter than that of the sensing electrode contact:

$$S(t)\approx \eta_{\tau }v\int V(t^{\prime })dt^{\prime }\cdot \Phi^{\prime \prime }(vt),$$ (8)

which is proportional to the recording electrode activating function [33] at the propagating location of the AP (i.e., *S*(*t*) does not reveal information about the its shape). In summary, unless the characteristic length scales of the recording electrode potential and the AP are comparable, zeroth and first order contributions vanish, and only small second order contributions remain. This is a consequence of the cancellation of the positive and negative lobes of $V^{\prime \prime }$, respectively $\phi^{\prime \prime }$.

#### 3.1.2. Fiber population:

Furthermore, additional signal cancellation occurs on the fiber population level, as the SFAP contributions consist of positive and negative lobes that mostly cancel out when integrated over time (apparent, e.g., when integrating over Eq (7) or (8)). The fiber-diameter dependent conduction velocity leads to shifted SFAP arrival times, such that low-order eCAP contributions from fiber populations with smoothly varying diameters cancel. Higher order, diameter-dependent contributions such as degree of recruitment, diameter probability density, and signal contribution scaling are primarily responsible for the eCAP signal. This can make the eCAP signal complex to interpret and sensitive to details of the topological fiber distribution and nerve anatomy.

We have shown that in many fascicles, a significant fraction of fibers, with small diameters, are not recruited (Fig 7C–7D). In most fascicles, the recruitment curve is steep (Fig 7D). Because conduction velocity is proportional to fiber diameter, SFAPs from smaller diameter fibers arrive later than those of larger diameter fibers. When there is a sharp cutoff in the diameter of the recruited fibers, the SFAP contributions of the activated fibers above the cutoff are not compensated anymore. This leads to large-amplitude deflections in the portion of the eCAP associated with the arrival of APs from the fibers immediately above the cutoff (Fig 7E), which in turn results in non-monotonicity in the peak-to-peak amplitude of the eCAP as a function of stimulus current (Fig 8). To our knowledge, the impact of partial fascicular recruitment, have not been previously reported and critically influences the interpretation of the eCAP.

#### 3.1.3. Inference of fiber properties.

We optimized the shape and scale parameters of the diameter distribution of myelinated afferent fibers in our model, while keeping those of efferents fixed, in order to minimize the difference between the modeled eCAP and a reference eCAP. Doing so, we were able to recover the parameters of the reference distribution (Fig 12). This example serves as a proof-of-concept for the use of similar optimization procedures on our model to recover fiber parameters from *in vivo* eCAP measurements. Such an approach may be useful in the development of personalized stimulation protocols, which may be able to target specific fiber populations, thus enhancing the effectiveness of VNS and minimizing side-effects.

When applying the procedure to *in vivo* data, it will naturally be necessary to optimize additional parameters, such as the fiber diameter statistics of all subpopulations. The necessity to consider a larger parameter space, as well as uncertainty about the model geometry, tissue properties, electrode contact, etc., complicate the optimization and could render the problem degenerate—multiple combinations of parameter values could lead to equally good solutions. This problem could be mitigated by the use of additional optimization criteria, such as, for example, eCAPs recruited at different stimulus current levels or measured from different pairs of electrodes.

### 3.2. Limitations and issues

#### 3.2.1. Extended reciprocity theorem:

The only assumption underlying the derivation of the extended reciprocity theorem is that the totality of the current sources cancel (i.e., charge is conserved). Besides the physical justification for that condition, it is also evident that dropping this condition results in a lack of well-definedness, as the measurable signal would become dependent on the chosen electric potential Gauge (i.e., $\phi (x)\to \phi (x)\;+\;\phi_{0}$ changes *S*(*t*)).

By computing the current source density as proportional to the second derivative of the transmembrane potential $V_{x}$, its integral becomes proportional to the difference between the spatial gradient of $V_{x}$ at the two terminals. If non-physical boundary conditions are used in the electrophysiology simulations or the multiplying potential *ϕ* is non-zero where the fiber model is truncated, care must be taken to include corresponding sinks to balance the current in the model.

#### 3.2.2. Semi-analytical eCAP model:

The semi-analytical nerve eCAP model makes several assumptions that may limit its applicability. We assume that the temporal shape of the AP is independent of diameter. While this assumption has been validated for the fiber models used in this study (Fig 2C–2D), it may not hold *in vivo*. We further assume that there is no systematic or stochastic background nerve activity. The model must also be refined, when complex stimulation paradigms are applied, such as multi-polar pulse-shapes, pulse-trains with high repetition rates, or high stimulation intensities that result in conduction blocking and non-monotonous recruitment curves. Assuming a reciprocal dependence of stimulation thresholds on fiber diameter is not uniformly correct for all fiber types and stimulation exposure lengths [34]. When it fails to hold, recruitment curves must instead be determined for a population of fibers representative of their real distribution. We find that applying a Gaussian jitter to the recruitment threshold for each fiber smooths the eCAP (Section B in S1 Text, Fig A in S1 Text).

For myelinated fibers, transmembrane current is mostly restricted to Ranvier nodes. When the exposing potential *ϕ* varies significantly over the length-scale of inter-node distances, it could become necessary to account for the spatial discreteness of the transmembrane current sources (incl. their stochastic position variability) and to replace the second derivative in $i=\eta_{\tau }\frac{\partial^{2}V_{x}(l)}{\partial l^{2}}$ by its finite-difference equivalent, while adapting $\eta_{\tau }$ to compensate for the reduced current source extent.

The finite element model assumes perfect contact between the recording electrodes and the nerve, and does not account for capacitive effects or scar tissue formation due to the insertion of a foreign body. The generalizability of the finite element model is also limited by uncertainty about the dielectric tissue properties and the perineurium thicknesses.

In principle, a too-coarse sampling of fiber diameters would limit the validity of our results. However, we show that doubling the sampling density, from 2000 to 4000 discrete diameters, hardly affects the signal (Section C in S1 Text, Fig B in S1 Text).

#### 3.2.3. Nerve topology differences between simulation and measurements and inter/intra-subject variability:

In view of the sensitive dependence of the eCAP signal on details of the topological fascicle and fiber distribution, as well as the nerve anatomy, it is important to keep in mind that the porcine histological data was not acquired from the same animal as the eCAP data. Thus, important differences can be expected, complicating validation. In view of future therapeutic application, this raises the problem of how to deal with important inter/intra-subject variability of the vagus topology. The following approaches appear to be relevant: 1) include minimal inter/intra-subject variability in the criteria for implantation site selection, 2) develop non-invasive means of characterizing subject- and implantation-site-specific topologies and fiber distributions (potentially by analyzing eCAP or electrical impedance tomography (EIT) signals; c.f. [35]), or 3) leverage closed-loop control strategies to compensate for the response prediction uncertainty.

#### 3.2.4. Impact of fiber model selection:

In this work, we have used a single fiber model to represent both myelinated efferents and myelinated afferents. In reality, afferent and efferent fibers can have different properties, including action potential shapes and stimulus thresholds [36]. Given that sensory fibers have lower thresholds than motor fibers [36], and given that sensory and motor fibers are largely segregated in different fascicles, we expect that if we modeled these differences explicitly, then for a given current level, we would be more likely to observe differing degrees of activation of primarily sensory fascicles and primarily motor fascicles.

It is, however, difficult to test this prediction *in silico*, as the models of sensory and motor fibers described in [36] are valid only for fiber diameters above 4 *μ*m, whereas a significant portion of myelinated fibers in our model are smaller than 4 *μ*m. While these models have been extended to smaller-diameter fibers [16], this extrapolation has not been extensively validated against *in vivo* data.

### 3.3. Comparison with previous work

Our model differs from that of Pe na et al. [25] in key aspects: 1) It considers fiber recruitment and accounts for partial activation, which is revealed to be crucial in interpreting eCAP signals. 2) It studies multi-fascicular porcine VNs, rather than the monofascicular rabbit nerve. 3) It is amenable to analytical evaluation, which permits the establishment of the relationship between signal features and parameters of interest. A technical differences is our use of simulated transmembrane voltages vs. transmembrane currents, which avoids separate computations for different fiber diameters. In conclusion, these developments allow for application to complex, multifascicular nerves and neurovascular bundles, and avoid resorting to problematic simplifications, such as neglecting the diameter dependence of partial fascicular activation.

### 3.4. Future directions

We anticipate that our model can be used to solve the inverse problem of reconstructing the distribution of fiber diameters in a nerve. We have already shown that optimization can be used to recover the diameter distribution of a fiber population (Fig 12), thus demonstrating, at least in principle, that optimizing our model to match a ground-truth eCAP can reveal information about the underlying structure of the nerve. While the overall distribution of diameters is known, the relative fractions of each fiber type in each fascicle cannot be known for an individual. However, if the location of the fascicles can be reconstructed, for example using EIT, the fiber proportions in each fascicle might be obtained by optimizing the agreement between measured and modeled eCAP signal. Knowledge of the fiber type distribution over fascicles for an individual greatly facilitates the interpretation of eCAPs signals and could help maximize stimulation selectivity and targeting, e.g., in treatment planning.

## 4. Conclusions

We have developed an extended reciprocity theorem approach suitable for the efficient computation of signals resulting from spatially extended electrophysiological activity in complex anatomical and dielectric environments. It has been used to establish a simplified analytical SFAP model—revealing interesting differences between myelinated fiber and unmyelinated fiber SFAP behavior—and a semi-analytical eCAP model. Employing these modeling approaches, simulations of VNS and recording were performed that leverage histological information about the multifascicular VN anatomy and its fiber population statistics. The impact on eCAP shape and magnitude of varying stimulation amplitude and stimulus and recording electrode configuration was studied. The eCAP signal is revealed to be more complex than commonly assumed, because of factors such as cancellation effects on the single fiber and fiber population level, and partial fiber recruitment, causing strong signal contributions. This complexity on the one hand complicates signal interpretation, but on the other hand might offer the opportunity to maximize signal information content by adapting the eCAP recording setup to increase the sensitivity to the quantity- or features-of-interest. The analytical and semi-analytical model are valuable instruments for that purpose, as they help understand the impact of electrode geometry (e.g., contact separation, dimension, orientation, mono- vs. bipolar, location) on eCAP recordings. The ratio of the width of the AP to the extent of the potential associated with the electrode is revealed to be a key parameter.

Our methods can be used to rapidly assess new stimulation and recording setups involving complex nerves and neurovascular bundles, e.g., for closed-loop control in bioelectronic medicine applications. By optimizing model parameters to minimize error between simulated and reference eCAPs, our methods can also be used to recover information about nerve structure and functional organization based on recorded eCAP signals.

## 5. Methods

### 5.1. Ethics statement

The protocol for all animal studies (no. 76/2014 PR) was approved by the Animal Care Committee of the Italian Ministry of Health and complied with Italian law (D.lgs. 26/2014).

### 5.2. Mathematical model

#### 5.2.1. Extended reciprocity theorem:

The well known reciprocity theorem [13] states that the voltage signal *S* recorded between two electrodes resulting from a dipolar current source $\vec{d}$ is related to the electric (E-)field generated at the dipole location $\vec{E}$ by (virtually) applying a current *J* to the same electrodes:


$$J\cdot S=-\vec{E}\cdot \vec{d}$$ (Eq 1 revisited)

Consequently, simulation-based predictions of the signals generated by a series of time-varying dipole sources ${\vec{d}}_{j}$ at locations *x*_*j*_ only require a single EM simulation of current applied to the sensing electrodes to evaluate $S(t)=-\sum_{j}({\vec{d}}_{j}\;\cdot \;\vec{E}(x_{j}))/J$.

We represent a neural fiber as a line with a transmembrane current density *i*(*l*) along its length. For simplicity, we will first consider discretized point current sources *I*_*i*_, which can either represent the integrated current density $i(l_{j})\;\cdot \;\Delta l$ over the *i*-th line segment of length Δl, or the current leaving a Ranvier node separated by a distance Δl from the next unmyelinated location on a myelinated fiber. It can be proven by induction that placing small dipolar sources ${\vec{d}}_{j}=-{\vec{\Delta l}}_{j}\sum_{k=1}^{j}I_{k}$ between the locations *x*_*i*_ and *x*_*i* + 1_ of two consecutive current sources separated by ${\vec{\Delta l}}_{j}=x_{j+1}-x_{j}$ reproduces the series of current sources *I*_*j*_ (charge conservation ensures that the last current source at the terminal is correctly obtained). Applying the reciprocity theorem, we get:


$$S=\sum_{j}{\vec{\Delta l}}_{j}\left[\sum_{k=1}^{j}I_{k}\right]\cdot \vec{E}\left(\frac{x_{j+1}+x_{j}}{2}\right)/J=\sum_{j}{\vec{\Delta l}}_{j}\cdot \vec{E}\left(\frac{x_{j+1}+x_{j}}{2}\right)\left[\sum_{k=1}^{j}I_{k}\right]/J,$$

which in the continuum limit becomes:


$$S=\frac{1}{J}\int_{0}^{l}E_{\text{tan}}(l^{\prime })\left(\int_{0}^{l^{\prime }}i(l^{\prime \prime })dl^{\prime \prime }\right)dl^{\prime },$$

where the tangential field $E_{\text{tan}}=\vec{E}\;\cdot \;\vec{\Delta l}/|\vec{\Delta l}|$. Using integration by parts we obtain:


$$S=\frac{1}{J}{\left[\left(\int_{0}^{l^{\prime }}E_{\text{tan}}(l^{\prime \prime })dl^{\prime \prime }\right)\left(\int_{0}^{l^{\prime }}i(l^{\prime \prime })dl^{\prime \prime }\right)\right]}_{l^{\prime }=0}^{l}-\frac{1}{J}\int_{0}^{l}\left(\int_{0}^{l^{\prime }}E_{\text{tan}}(l^{\prime \prime })dl^{\prime \prime }\right)i(l^{\prime })dl^{\prime }$$

Using the definition of the electric potential, $\phi (l)=\phi (0)-\int_{0}^{l}E_{\text{tan}}dl^{\prime }$:


$$S=-\frac{1}{J}{\left[\phi (l^{\prime })\left(\int_{0}^{l^{\prime }}i(l^{\prime \prime })dl^{\prime \prime }\right)\right]}_{l^{\prime }=0}^{l}+\frac{1}{J}\int_{0}^{l}\phi (l^{\prime })i(l^{\prime })dl^{\prime },$$

Because of current conservation, $\int_{0}^{l}i(l^{\prime })dl^{\prime }=0$ and the expression reduces to:


$$S=\frac{1}{J}\int_{0}^{l}\phi (l^{\prime })i(l^{\prime })dl^{\prime }$$

With Φ=ϕ/J, we obtain the *extended reciprocity theorem:*


$$S=\int_{0}^{l}\Phi (l^{\prime })i(l^{\prime })dl^{\prime }$$ (Eq 2 revisited)

We call Φ(l) the “sensitivity function”.

#### 5.2.2. Analytical SFAP model:

From the extended reciprocity theorem, it follows that the SFAP is the convolution in space of the transmembrane current with the extracellular potential along the neuron:


S(t)=∫i(l−tv)Φ(l)dl (Eq 3 revisited)

By Ohm’s law, a neuron’s axial current *I* is linked to its membrane potential by $\frac{\partial V}{\partial x}=-Ir_{i}$, where *r*_*i*_ is the axial resistance per unit length. Taking the derivative with respect to *x*, $\frac{\partial^{2}V}{\partial x^{2}}=-r_{i}\frac{\partial I}{\partial x}$. By current conservation, $\frac{\partial I}{\partial x}=-i$, where *i* is the transmembrane current per unit length. Thus, $\frac{\partial^{2}V}{\partial x^{2}}=r_{i}i$. Intracellular resistivity *ρ* is related to *r*_*i*_ by $r_{i}=\frac{4\rho }{\pi d^{2}}$, where *d* is neuronal diameter. Thus, the trans-membrane current per unit length for a particular fiber type (index _τ_) can be obtained as $i=\eta_{\tau }\frac{\partial^{2}V_{x}(l)}{\partial l^{2}}$, where $\eta_{\tau }=\frac{\pi d^{2}\sigma_{\tau }}{4}$, $\sigma_{\tau }=\frac{1}{\rho }$ is the axial conductance of an axon (which is assumed to be constant over the length of the axon), $V_{x}$ is the transmembrane voltage (distinguished by the subscript *x* from the temporal transmembrane potential $V_{t}(t)$), and *d* is its diameter ([37,38], see Sect 3.2.2 regarding myelinated fibers).

Thus,


$$S(t)=\eta_{\tau }\int \frac{\partial^{2}V_{x}(l-vt)}{\partial l^{2}}\Phi (l)dl$$

where Φ is the extracellular potential generated by the recording electrodes, normalized to the applied current, and *v* is the conduction velocity.

If $V_{x}(x)$ is the transmembrane potential along the fiber, then, for $V_{x}$ at *t* = 0, $V_{x}(x)=V_{t}(-\frac{x}{v})$, where $V_{t}(t)$, obtained at *x* = 0, is the profile of the AP in time that has the benefit of only being weakly fiber diameter dependent. For convenience, we define *t* = 0 to coincide with the peak of $V_{t}$. Thus, $V_{x}(x-vt)=V_{t}(t-\frac{x}{v})$, where the important diameter dependence enters through *v*(*d*). With a change of variables $x=t^{\prime }\;\cdot \;v$, we can express this convolution in the time domain:


$$S(t)=\frac{\eta_{\tau }}{v^{2}}\int \frac{\partial^{2}V_{t}(t-t^{\prime })}{\partial t^{\prime 2}}\Phi (v\cdot t^{\prime })d(v\cdot t^{\prime })=\frac{\eta_{\tau }}{v}(\partial_{t}^{2}V_{t}(t)*\Phi (v\cdot t))$$ (Eq 4 revisited)

where ‘*’ signifies convolution in time.

#### 5.2.3. Verification:

We verify our implementation of the analytical model of the SFAP as follows: First, we compare the results of the analytical SFAP calculation to the point source approximation. In Sim4Life, we generate a block sized 0.2m×0.2m×0.5m. A model of a rat sciatic nerve fiber [31] with diameter 4 *μ*m and length 300 mm is placed inside the block. Two spherical recording electrodes, each with radius 1 mm, representing point electrodes, are placed inside the block. AN LF-EM QS simulation was executed, applying Dirichlet boundary conditions of ±1V to the recording electrodes. In postprocessing, the current applied between the electrodes is calculated by interpolating current flux over a virtual sphere surrounding one of the electrodes.

Using the Sim4Life T-NEURO module, an action potential was triggered in the fiber model by an IClamp Point Process, with amplitude 10 nA and duration 0.2 ms, placed in internode 0. Transmembrane currents and potentials at all segments were recorded at 100 evenly-spaced sampling times using an Overall Line Sensor. The T-NEURO simulation was run for 30 ms with a time step of 0.0025 ms. For each of the two electrodes, the SFAP was calculated using the point-source approximation $V=\frac{1}{4\pi \sigma_{e}}\Sigma_{i}\frac{{\text{i\_membrane}}_{i}(t)}{\left|{\vec{r}}_{i}-{\vec{r}}_{e}\right|}$, where $\Sigma_{i}$ refers to a summation over all compartments in the model, ${\vec{r}}_{i}$ is the location of the *i*-th compartment, and ${\vec{r}}_{e}$ is the location of the electrode. The difference between the two potentials was calculated and compared to the SFAP calculated using Eq 4.

Next we compare the results of the analytic SFAP model to those of the Sim4Life Neural Sensing Tool. In Sim4Life, we place a model of a rat sciatic nerve fiber [31], 100 mm in length and 20 *μ*m in diameter, in the center of a cylindrical volume of the same length and 2.5 mm in diameter. Two crescent-shaped recording electrodes, with a length 2 mm and thickness 0.5 mm, are placed around the cylinder. The recording electrodes are separated by 10 mm, and the proximal electrode is 60 mm from the start of the nerve fiber. AN LF-EM QS simulation was executed as described above.

A neural simulation was conducted as described previously. Transmembrane currents and potentials at all segments were recorded at 100 evenly-spaced sampling times for 2 ms using an Overall Line Sensor. The SFAP was calculated using Sim4Life’s Neural Sensing tool, and compared to the SFAP calculated using Eq 4.

#### 5.2.4. Gaussian approximation:

We simplify the analytical model in Sect 2.2 by approximating the shape of the action potential and of the sensitivity function as Gaussians. The AP shape $V_{t}(t)$ is parameterized as $V_{t}(t)=V_{p}\exp\left(-\frac{t^{2}}{2w_{V}^{2}}\right)$ and the potential near a local monopolar electrode Φ(x) is parameterized by $\Phi (x)=\Phi_{p}\exp\left(-\frac{(x-x_{c}{)}^{2}}{2w_{\phi }^{2}}\right)$. The constants $V_{p}$, $w_{V}$, $\Phi_{p}$ and $w_{\phi }$ define the amplitude and the width of these Gaussians. We note that these approximations are used only for the analytical investigation described in this section in order to allow for a closed-form solution to the SFAP, and not for the semi-analytical investigations described elsewhere. A bipolar electrode can be approximated as the combination of two monopolar contacts: $\Phi_{E}(x;x_{c,1},w_{\phi ,1},\Phi_{p,1})-\Phi_{E}(x;x_{c,2},w_{\phi ,2},\Phi_{p,2})$.

Taking the time Fourier transform ${ℱ}_{t}(\;)$ of Eq 4, we obtain


$${ℱ}_{t}\left(S(t)\right)(\omega )=-\frac{\eta_{\tau }\omega^{2}}{v^{2}}{ℱ}_{t}(V)(\omega )\cdot {ℱ}_{x}(\Phi )(\omega /v)$$

and through inverse Fourier transform ${ℱ}_{t}^{-1}(\;)$ we recover the SFAP.

Using the above equation, and assuming (monopolar) Gaussian shapes for $V_{t}$ and Φ, we obtain:


$$S(t)=\frac{V_{p}\Phi_{p}\eta_{\tau }w_{\phi }w_{V}}{w_{C}^{5}v^{2}}e^{-\frac{{\left(\frac{x_{c}}{v}-t\right)}^{2}}{2w_{C}^{2}}}\left(t-\left(\frac{x_{c}}{v}+w_{C}\right)\right)\left(t-\left(\frac{x_{c}}{v}-w_{C}\right)\right),$$ (Eq 5 revisited)

where $w_{C}=\sqrt{(\frac{w_{\phi }}{v}{)}^{2}+w_{V}^{2}}$ is the temporal Gaussian width after convolution and evidently the characteristic time-scale of the recorded signal. The SFAP shape corresponds to the typical peak between two side-lobes of opposite polarity—a result of the second derivative in Eq 4—that peaks at $x_{c}/v$—the time it takes to reach the recording electrode. The peak magnitude is:


$$\frac{V_{p}\Phi_{p}\eta_{\tau }w_{\phi }w_{V}}{v^{2}w_{C}^{3}}.$$

Two limit cases of the Gaussian approximation model are of interest: When $v\gg w_{\phi }/w_{V}$ (i.e., the AP extent is much larger than the region to which the sensing electrode is sensible), the temporal width $w_{C}\approx w_{V}$ and the amplitude becomes $\frac{V_{p}\Phi_{p}\eta_{\tau }w_{\phi }}{v^{2}w_{V}^{2}}$. When $v\ll w_{\phi }/w_{V}$, the temporal width $w_{C}\approx w_{\phi }/v$ and the amplitude becomes $\frac{V_{p}\Phi_{p}\eta_{\tau }vw_{V}}{w_{\phi }^{2}}$ (Table 1). For the recording electrodes used in this paper, $w_{\phi }\sim 1$ mm (Fig 4A). For the myelinated fiber model used in this study, $v\sim 4.3\;\cdot \;10^{6}d$ and $w_{V}\sim 5\;\cdot \;10^{-5}$s (Fig 2A, 2C). For the unmyelinated fiber model, $v\sim 470\sqrt{d}$ and $w_{V}\sim 2\;\cdot \;10^{-4}$s (Fig 2B, 2D). Thus, for the unmyelinated fiber model, realistic fiber diameters are in the $v\ll w_{\phi }/w_{V}$ regime—the theoretical diameter *d*  at which $v=w_{\phi }/w_{V}$ is >100 *μ*m. For myelinated fibers, however, $d^{*}\approx$4.7 *μ*m, which is comparable in scale to the diameters of the relevant fiber population, such that their eCAP contribution show a more complex *d* dependence in terms of magnitude, but also shape (see Sect 3.1).

#### 5.2.5. Limit cases:

For propagating APs, Eq 3 can be expressed as:


$$S(t)=\frac{\eta_{\tau }}{v^{2}}\int {V_{t}^{\prime }}^{\prime }(t-\frac{x}{v})\Phi (x)dx=\eta_{\tau }\int V_{t}(t-\frac{x}{v})\Phi^{\prime \prime }(x)dx$$ (Eq 6 revisited)

If the AP’s spatial extent is much larger than that of a sensing electrode contact at location *x*_0_ (or, more precisely, that of its Φ along the fiber; see as well Sect 2.3), $\int V_{t}(t\;-\;\frac{x}{v})\Phi^{\prime \prime }(x)dx$ can through Taylor expansion be approximated to second order as:

$\int \left(V_{t}(t-\frac{x_{0}}{v})-\frac{\left(x-x_{0}\right)}{v}V_{t}^{\prime }(t-\frac{x_{0}}{v})+\frac{(x-x_{0}{)}^{2}}{2v^{2}}{V_{t}^{\prime }}^{\prime }(t-\frac{x_{0}}{v})\right)\Phi^{\prime \prime }(x)dx=$

$\left(V_{t}(t-\frac{x_{0}}{v})+\frac{x_{0}}{v}V_{t}^{\prime }(t-\frac{x_{0}}{v})+\frac{x_{0}^{2}}{2v^{2}}{V_{t}^{\prime }}^{\prime }(t-\frac{x_{0}}{v})\right)\int \Phi^{\prime \prime }(x)dx$ ...

$-\left(\frac{1}{v}V_{t}^{\prime }(t-\frac{x_{0}}{v})+\frac{x_{0}}{v^{2}}{V_{t}^{\prime }}^{\prime }(t-\frac{x_{0}}{v})\right)\int x\Phi^{\prime \prime }(x)dx+\frac{1}{2v^{2}}{V_{t}^{\prime }}^{\prime }(t-\frac{x_{0}}{v})\int x^{2}\Phi^{\prime \prime }(x)dx$.

This is a common case for myelinated fibers. By partial integration:


$$\int \Phi^{\prime \prime }(x)dx=\Phi^{\prime }(x_{e})-\Phi^{\prime }(x_{s}),$$

$$\int x\Phi^{\prime \prime }(x)dx=x_{e}\Phi^{\prime }(x_{e})-x_{s}\Phi^{\prime }(x_{s})-\Phi^{\prime }(x_{e})+\Phi^{\prime }(x_{s}),$$

$$\int x^{2}\Phi^{\prime \prime }(x)dx=x_{e}^{2}\Phi^{\prime }(x_{e})-x_{s}^{2}\Phi^{\prime }(x_{s})-2x_{e}\Phi (x_{e})+2x_{s}\Phi (x_{s})+2\int \Phi (x)dx$$

At sufficiently remote terminals *x*_*s*_ and *x*_*e*_, $\Phi^{\prime \prime }(x_{e})=\Phi^{\prime }(x_{e})=\Phi^{\prime }(x_{s})=\Phi^{\prime }(x_{s})=0$ and $\Phi (x_{e})=\Phi (x_{s})=\Phi_{\infty }$, such that *S*(*t*) reduces to:


$$S(t)\approx \frac{\eta_{\tau }}{v^{2}}{V_{t}^{\prime }}^{\prime }(t-\frac{x_{0}}{v})\int (\Phi (x)-\Phi_{\infty })dx=\int (\Phi (x)-\Phi_{\infty })dx\cdot i(t-\frac{x_{0}}{v})$$ (Eq 7 revisited)

For the converse case, when the spatial extent of the AP is much shorter than that of the electrode potential, a similar analysis shows that $\int {V_{t}^{\prime }}^{\prime }(t-\frac{x}{v})\Phi (x)dx$ can be approximated as:


$$S(t)\approx \eta_{\tau }v\int V(t^{\prime })dt^{\prime }\Phi^{\prime \prime }(vt)$$ (Eq 8 revisited)

To obtain this result, we substitute $x=v\;\cdot \;t^{\prime }$, expand $\Phi^{\prime \prime }(x)$ around v·t to second order, and consider that ${V_{t}^{\prime }}^{\prime }(x_{e})=V_{t}^{\prime }(x_{e})=V_{t}(x_{e})=V_{t}^{\prime }(x_{s})=V_{t}^{\prime }(x_{s})=V_{t}(x_{s})=0$.

#### 5.2.6. Semi-analytical eCAP model:

In a real nerve, a large number of fibers are simultaneously active. To determine the resulting eCAP signal, their SFAP contributions must be combined. Because of the linearity of Maxwell’s equations, simple additive combination can be used. To avoid simulating SFAPs from each fiber in the nerve, we construct the eCAP based on a representative SFAP per fiber type (index _τ_): myelinated afferents and efferents, and unmyelinated afferents and efferents. The representative SFAP is interpolated from a neuronal simulation as described in Sect 5.4.3; the Gaussian approximation described in Sect 5.2.4 is not used in the semi-analytical model. Electrophysiologically, afferents and efferents are assumed to be identical (but c.f. Sect 3.2.4).

For each fascicle (index _*i*_), we consider 2000 fibers of each fiber type, with uniformly spaced diameters from 0.1 to 15 *μ*m. Each SFAP is scaled by the fiber diameter density $n_{i,\tau }(d)=N_{i,\tau }\;\cdot \;p_{\tau }(d)$ ($N_{i,\tau }$: number of fibers of the given type, $p_{\tau }(d)$: diameter probability distribution) and the degree of fascicle recruitment $R_{i,\tau }(d)$ (Fig 5A–5B). Note that $R_{i,\tau }(d)$ implicitly depends on the stimulation magnitude and pulse shape.

The fiber number $N_{i,\tau }$ is a random variable drawn from distributions (obtained by [19] based on *in vivo* data) which are dependent on the location of the fascicle within the nerve (myelinated afferents and efferents are localized on opposite sides of the nerve, and unmyelinated efferents are colocalized with myelinated afferents), and scaled by the area of the fascicle. For details on the calculation of $R_{i,\tau }$, see Sect 5.4.3.

In multifascicular nerves, the different fascicles are separated by semi-insulating perineurium, such that within fascicle *i* the $\phi_{i}(l)$ along fiber trajectories is similar for all fibers, but the eCAP contributions must be computed using different $\phi_{i}(l)$ for the different fascicles (Fig 5E).

Thus, the eCAP is given by

$$S(t)=\Sigma_{\tau }\Sigma_{i}\partial_{t}^{2}V_{t}(t)*X_{i,\tau }(d,t),$$ (9)

where $\partial_{t}^{2}V_{t}(t)$ is the second temporal derivative of the transmembrane potential during the action potential, $X_{i,\tau }(d,t)$ is the fascicle “exposure function”, defined as $\Sigma_{d}R_{i,\tau }(d)\;\cdot \;n_{i,\tau }(d)$ $\Delta d\;\cdot \;\frac{\eta_{\tau }}{v}(d)\;\cdot \;\Phi_{i}(d,t)$ and Δd is the width of the discretized fiber diameter bins (Section C in S1 Text). We assume that all recruited fibers fire at the same time, and that all action potentials are initiated at the same location along the fiber.

### 5.3. Experimental data acquisition

This study involved five healthy adult male Göttingen minipigs (Ellegaard Göttingen Minipigs A/S, Dalmose, Denmark; average body weight of 35 kg). The animals were premedicated with Zoletil (10 mg/kg) and Stressnil (1 mg/kg). Anesthesia was induced with propofol (2 mg/kg intravenously) and maintained with 1% to 2% sevoflurane in air enriched with 50% oxygen during mechanical ventilation [39][40]. To prevent dehydration, each animal received a continuous infusion of 500 mL of sodium chloride (0.9%) solution throughout the experiments. A longitudinal incision followed by a sternotomy was performed, and blunt dissection was used to expose the VN at both the cervical and thoracic levels.

The NeuHeart Vagal Regenerative Autonomic Interface (VRAI) electrode [27], which is a transversal, intrafascicular, multichannel electrode (TIME) featuring eight contacts on each side was inserted in the cardiac branch of the VN for stimulation purposes, and a cuff electrode with four ring contacts was placed at a distance of 6  cm from the stimulation electrode to record eCAP signals. Varying stimulation intensities (100 to 500 μA), active contacts, and pulse repetition rates (1 to 50 Hz) were applied. The experimental presented in this paper have all contacts active, and use a 50 Hz pulse repetition rate. Note that in our simulations, we do not model the effect of pulse repetition. Stimulus-triggered averaging was used to reduce noise in the *in vivo* eCAPs.

Differential recordings from the four channels commercial cuff electrode (World Precision Instruments, Sarasota, FL, USA) were acquired using a TDT system (Tucker Davis Technologies; Alachua, FL, USA), with a sampling rate of 24k samples/s. Each recording session was composed of 10 s baseline activity, followed by 1 min VNS, and 1 min of washout.

In this work, we use a bipolar recording configuration, in which we measure the differential signal between two cuff electrodes. Bipolar recordings correspond to the finite difference derivative of the corresponding monopolar recordings (Fig 11, Sect 2.6). As such, they contain more information than a single monopolar recording—the difference between two monopolar recordings (Fig 11) can increase the visibility of information about the diameters of activated fibers, which is reflected in the different shapes of the signals at the two contacts due to differences in propagation velocity. The use of a bipolar recording configuration also reduces noise in the signal, by allowing for common-mode rejection (artifacts, such as EMG activity, which are detected at identical amplitudes at both contacts, do not appear in the differential signal because they cancel out).

### 5.4. Realistic, multifascicular nerve model

To demonstrate the application of the extended reciprocity theorem and of the semi-analytical modeling in a realistic setup, we modeled the experimental setup from Sect 5.3.

#### 5.4.1. Anatomical and electrode geometry:

A 2D cross section of the pig VN was segmented from a histology slice, a model of the stimulating VRAI and the recording cuff electrode was integrated, and the tissue contours were extruded to produce a 2.5D model.

We simulate two different orientations of the stimulation electrode, one in which the electrode is oriented along the major axis of the nerve (Fig 1D) and one in which it is oriented along the minor axis (Fig 1E). The geometry of the VRAI is somewhat simplified for computational efficiency: the shape of the VRAI is simplified to a rectangle outside of the nerve; the return electrode is moved down the shank of the substrate, closer to the nerve, and the contacts are modeled as squares rather than circles. To simulate the electrode placement along the major axis of the nerve, the insulating substrate is excluded, in order to prevent the electrode from piercing any of the fascicles. As all of the contacts, on both sides of the electrode, are active in our simulation, the exclusion of the substrate does not have a significant effect on the resulting E-field.

The cuff electrode consists of four contacts, each with a thickness of 125 *μ*m, and with 3mm inter-contact spacing. The contacts are surrounded by an insulating material. Both the contacts and the insulation extend around the majority of the circumference of the nerve; the entire area of the cuff is used as a recording contact (Fig 1F). In addition to the large recording electrode, we also, separately, simulate two configurations in which the recording electrodes cover a much smaller portion of the nerve circumference (Fig 10C–10D); the position of the recording electrode is changed between these two configurations.

For reasons of computational efficiency, the stimulating and recording electrodes are simulated separately, on the basis of the assumption that the presence of the recording electrode does not have a significant effect on the electric fields produced by the stimulating electrode, and vice versa.

#### 5.4.2. EM modeling:

The complete model was discretized, and the Sim4Life Electro-Ohmic Quasi-Static (EQS) solver was used to calculate potentials and E-fields resulting from an electrical stimulus applied at the VRAI electrodes and from a virtual current applied at the cuff electrodes.

The finite element simulation solves the equation ∇σ∇ϕ=0, where *σ* is the electrical conductivity distribution and Φ is the electric potential, from which the E-field is obtained as $\vec{E}=-\nabla \phi$ and the current density as $\vec{j}=\sigma \vec{E}$. This approximation is suitable, as ohmic currents dominate over displacement currents and the simulation domain is much smaller than the wavelength in tissue [41]. Material properties were assigned to all tissues and materials according to Table 2, including the anisotropy of the intrafascicular endoneurium (tensorial *σ*). Thin resistive layer settings were assigned to patches at the interfaces between fascicles and the interfascicular tissue with a thickness computed as 3 % of the fascicle’s equivalent diameter [42].

**Table 2:** Tissue conductivities. For anisotropic endoneurium, the longitudinal and transversal conductivities are listed.

Material	Conductivity (S/m)	Source
Endoneurium [l,t]	[0.083,0.57]	[42]
Muscle	0.355	Averaging of tissues from [43]
Silicone	10^−12^	[43]
Perineurium	0.0021	[42]
Epineurium	0.083	[42]

As described in Sect 5.2.6, the sensitivity function $\Phi_{i}(l)$ is calculated for each fascicle, by interpolating the potential field obtained from the finite element simulation along the fascicle center-line. The interpolated potential field is post-processed to ensure that it reaches 0 at the ends of the nerve. At each end of the $\Phi_{i}(l)$ curve, we identify the point having a predefined minimal slope. We linearize the Φ curve from this point, until the intersection with the line Φ=0, after which Φ is set to 0.

#### 5.4.3. Neuronal dynamics simulations:

The temporal profile of the AP was obtained from a multicompartmental simulation in Sim4Life of a rat myelinated sensory-motor neuron [31,44] and a Sundt unmyelinated neuron [32], for myelinated and unmyelinated fibers, respectively.

The recruitment *R*_*i*_ for a given stimulus waveform is calculated as follows: In our finite element model of the VN, we instantiate a population of myelinated fibers with uniform diameter $d_{0}=4\;\mu$m in each fascicle and a unmyelinated population with $d_{0}=0.8\;\mu$m. For each fascicle, a recruitment curve $R_{i}^{*}(I)$ was generated by titrating simulated VRAI field induced nerve activation. Titrations were performed using a single pulse, as the highest *in vivo* pulse repetition rate used in this study, 50 Hz, is well below the frequency at which pulse repetition rate influences threshold (c.f. Section A in S1 Text). Thanks to the ∼$\frac{1}{d}$ diameter-dependence of fiber stimulation thresholds, $R_{i}^{*}(I)$ can be recast as diameter-dependent *R*_*i*_(*d*) for a given stimulus of intensity *I* according to $R_{i}(d)=R_{i}^{*}(\frac{d\;\cdot \;I}{d_{0}})$.

Fiber electrophysiology simulations were executed within Sim4Life v8.0 using the T-Neuro module, which couples the EM solver with Yale’s NEURON [45]. Reference simulations to obtain shapes of the AP were performed with an axon diameter of 20 *μ*m for myelinated fibers and 0.8 *μ*m for unmyelinated fibers, using a time-step of 0.0025 ms. Simulations performed with myelinated and unmyelinated diameters of 10 *μ*m and 0.4 *μ*m, respectively,confirmed that the shape of the AP is independent of fiber diameter (Fig 2C-D). For neural titration simulations, $d_{0}=0.8\;\mu m$ for unmyelinated fibers and 4μm for myelinated fibers.

As discussed in Sect 5.2.6, SFAPs for each of the sampled diameters are rescaled and summed to synthesize the eCAP. Rescaling is appropriate, because the temporal AP shape $V_{t}$ only weakly depends on *d* (see Fig 2C–2D). For myelinated fibers, conduction velocity is proportional to fiber diameter $v_{m}(d)=a\;\cdot \;d$, while for unmyelinated fibers, it is proportional to the square root of fiber diameter $v_{m}(d)=b\sqrt{d}$ ([46,47]; see Fig 2A–2B).

### 5.5. Inverse problem solving

To investigate the suitability of the eCAP and our modeling approach for inferring fiber population statistics (diameter distribution), a proof-of-concept study was conducted. Using the semi-analytic model, we generate “ground-truth” eCAPs from known fiber diameter distributions. We then use the mesh_adaptive_search method in the Dakota software package to optimize the shape and scale parameters of the fiber diameter distribution of the myelinated afferent fiber subpopulation in our computational model, beginning with an arbitrary set of parameters. During optimization, Dakota iteratively calls our Python implementation of the *in silico* eCAP prediction for the assessed parameter configuration. The objective function to be minimized is the L1 norm between the *in silico* and reference signals: $\Sigma_{i}|V_{i}\;-\;\hat{V_{i}}|$, where *V* is the *in silico* signal, $\hat{V}$ is the reference signal, and the index *i* refers to each time step.

### 5.6. Implementation and code availability

The image-based nerve model construction was performed in Sim4Life v7.2 (ZMT Zurich MedTech AG, Switzerland) The EM simulations, fiber functionalization, electrophysiology simulations, and extraction of quantities-of-interest were performed in Sim4Life v8.0.

The semi-analytic model was run on a Windows desktop: parallelizing over 8 cores and using 64 GB of RAM, each simulation of the vagus nerve, for a single set of stimulus and recording parameters, requires 5 minutes. Finite element models were run on a Windows server with a 2.30 GHz, 32-core Xeon E5-4610 CPU and 512 GB RAM. Only one core was used. Neural simulations were run on a similar server, using 55 cores.

The finite element models used in this paper are accessible at https://osparc.io/#/study/e03935d2-79a5-11ef-8269-0242ac174ae0 and at https://zenodo.org/records/15848037. The code used to run the semi-analytic model and make the figures are available at https://github.com/joseph-tharayil/vagusNerve. The code used to run the optimization procedure is available at https://github.com/joseph-tharayil/vagusOptimization.

## Supporting information

S1 Text Investigations on model assumptions. (PDF)
