# Correction: Simulation insights on the compound action potential in multifascicular nerves

**DOI:** 10.1371/journal.pcbi.1013902

**Published:** 2026-01-20

**Authors:** The PLOS Computational Biology Staff

## Notice of republication

This article was republished on December 23, 2025, to correct an error in the first author’s name in the XML version of the article. The author’s name was incorrectly indexed as James Tharayil J. The correct name is Joseph James Tharayil, and the correct citation is: Tharayil JJ, Zinno C, Agnesi F, Lloyd B, Farcito S, Cassara A, et al. (2025) Simulation insights on the compound action potential in multifascicular nerves. PLOS Computational Biology 21(9): e1013452. https://doi.org/10.1371/journal.pcbi.1013452

The copyright statement in the XML version of the article has also been updated to include the corrected author name. These errors affected the article’s indexing in PubMed. The PDF version of the article was correct at the time of original publication. The publisher apologizes for the errors.
