# Supplementary material for: Simulation insights on the compound action potential in multifascicular nerves
Source: PLoS Comput Biol. 2025 Sep 12;21(9):e1013452. doi: 10.1371/journal.pcbi.1013452 (PMC12431235; doi:10.1371/journal.pcbi.1013452)
Supplement: S1 Text — (PDF) [file pcbi.1013452.s001.pdf]

Investigations on model assumptions

A Pulse repetition rate

After an initial evoked action potential, neurostimulation with inter-pulse intervals close to the duration of the refractory period will require larger-than-threshold currents to evoke subsequent action potentials [1]. At higher frequencies, electrical stimulation can abolish neural responses entirely, due to activation of K^+ channels or inactivation of Na^+ channels [2]. Our semi-analytical model does not account for either of these effects; we assume that a stimulus pulse reliably triggers action potentials for the stimulus frequencies ($\leq$50 Hz) used in the *in vivo* experiments in this study.

To determine if pulse repetition rates have a practical impact on stimulus threshold, we simulate the number of action potentials triggered by a train of pulses just above threshold, for various stimulus frequencies, and for two different fiber diameters, using the T-Neuro solver in Sim4Life. At pulse repetition rates of 100 Hz and 200 Hz, an action potential is reliably triggered by each pulse. At pulse repetition rates of 300 Hz, 400 Hz, and 500 Hz, only half of all pulses trigger action potentials. As the maximal *in vivo* pulse repetition rate used in this study is 50 Hz, we conclude that calculating thresholds using a single pulse is justified.

B Gaussian Jitter in Recruitment Curves

Our model assumes that the relationship between fiber diameter and recruitment is strictly monotonic. It does not account for variations in threshold due to ongoing neural activity, to inhomogeneity in the distribution of fiber diameters within the fascicle, or to uncertainty in the electromagnetic model. To estimate the effects of this uncertainty, we add, for each fiber in the neural titration simulation, a normally-distributed random variable to the threshold current, with mean of 0 and standard deviation equal to a particular fraction f of the threshold current. Then, as described in Section 5.3.3, the recruitment probability for each fiber diameter in the semi-analytic model is parameterized based on the threshold values (now including additive noise) from the titration. The process is repeated for $f \in \{0.1, 0.2, 0.3, 0.4\}$ The addition of noise to the thresholds effectively smooths the recruitment curves, such that fiber recruitment is more gradual than predicted by our original semi-analytic model.

When applying Gaussian jitter to the recruitment threshold for each fiber, the eCAP signal is smoothed, and peaks can even merge(Fig. A), reducing differences between the contributions of different fascicles, and thus reducing the signal information content.

C No Effect of Diameter Sampling Density on eCAP

To ascertain whether we have sufficient diameter sampling resolution in our implementation of Equation 9, we simulated eCAPs using either 2000 or 4000 diameter samples between 0.1 and 15 μm. The eCAPs are almost identical (Fig. B).

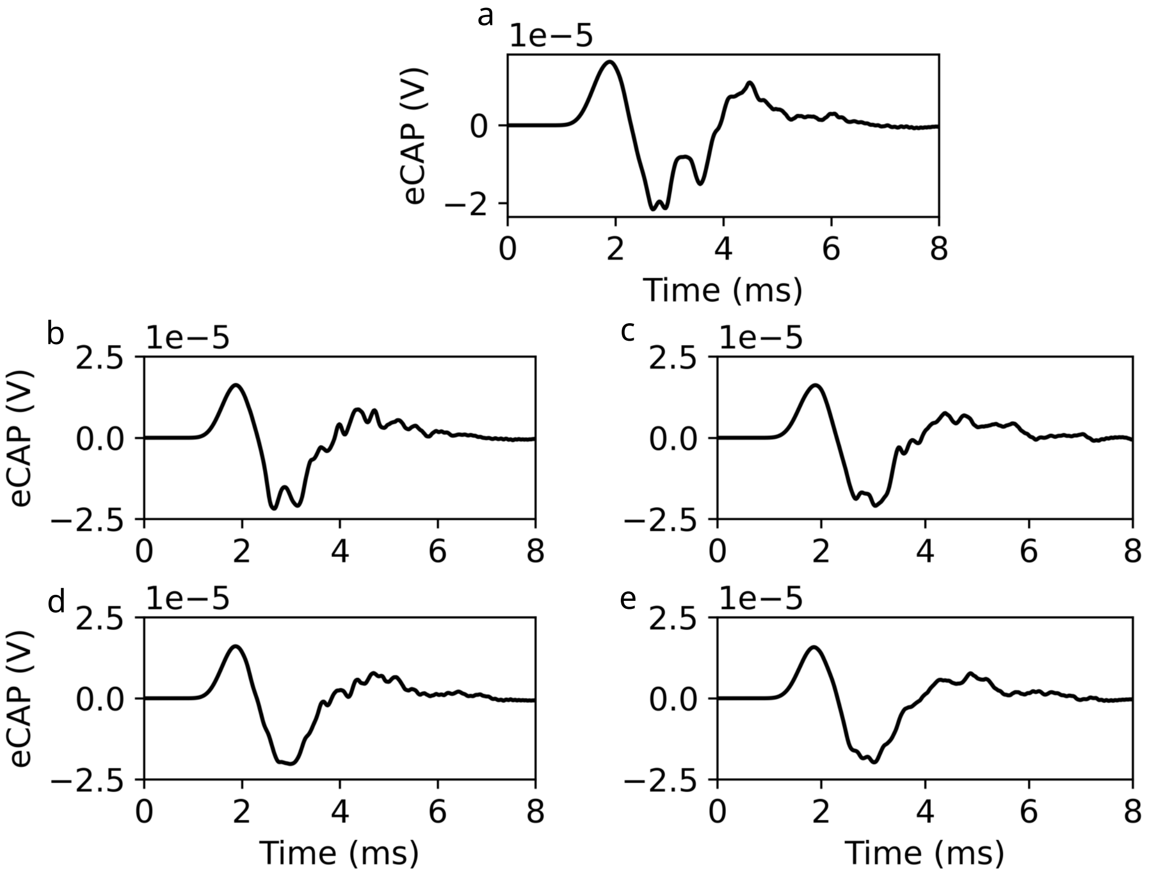

Figure A: a: eCAP produced at 31.25 μA stimulation current without adding Gaussian jitter to the recruitment curve. b-e: eCAPs produced at 31.25 μA with Gaussian jitter of 10%, 20%, 30%, and 40%, respectively, added to the recruitment curve.

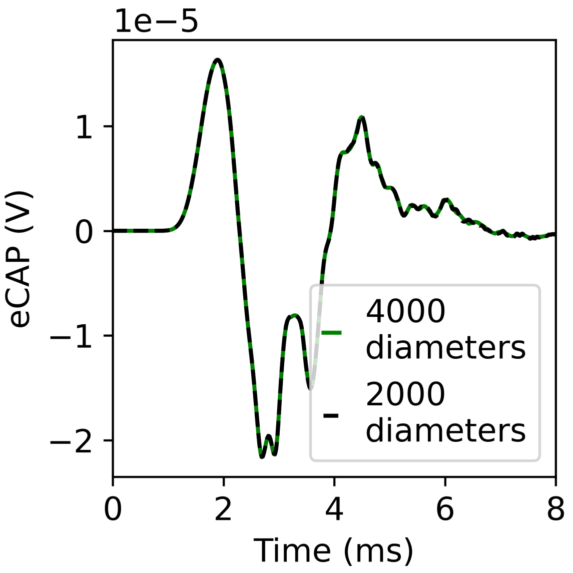

Figure B: Increasing the discretization resolution of the fiber diameter range considered in the eCAP computation from 2000 to 4000 hardly affects the modeling results.

References

[1] Y. Zhong, J. Wang, J. Beckel, W. C. de Groat, and C. Tai, “Model analysis of post-stimulation effect on axonal conduction and block,” *IEEE Transactions on Biomedical Engineering*, vol. 68, no. 10, pp. 2974–2985, 2021.

[2] J. Avendaño-Coy, D. Serrano-Muñoz, J. Taylor, C. Goicoechea-García, and J. Gómez-Soriano, “Peripheral nerve conduction block by high-frequency alternating currents: A systematic review,” *IEEE Transactions on Neural Systems and Rehabilitation Engineering*, vol. 26, no. 6, pp. 1131–1140, 2018.
